# Mothering, Substance Use Disorders and Intergenerational Trauma Transmission: An Attachment-Based Perspective

**DOI:** 10.3389/fpsyt.2019.00728

**Published:** 2019-10-18

**Authors:** Florien Meulewaeter, Sarah S. W. De Pauw, Wouter Vanderplasschen

**Affiliations:** Department of Special Needs Education, Ghent University, Ghent, Belgium

**Keywords:** mothers, substance use, children, attachment, trauma, intergenerational

## Abstract

**Background:** A growing body of research underlines that interpersonal trauma in childhood leads to heightened susceptibility for substance use disorders (SUDs) in later life. Little research has been conducted on parenting experiences of mothers in recovery from substance use, taking into account their own upbringing as a child and the potential aftermath of interpersonal childhood trauma.

**Methods:** Through in-depth qualitative interviews, 23 mothers with SUDs reflected on parenting experiences and parent-child bonding, related to both their children and parents. Interviews were transcribed verbatim and data were analyzed adopting thematic analysis.

**Results:** Throughout the narratives, consequences of trauma on mothers’ sense of self and its subsequent impact on parenting arose as salient themes. Five latent mechanisms of intergenerational trauma transmission were identified: 1) early interpersonal childhood trauma experiences in mothers; 2) trauma as a precursor of substance use; 3) substance use as a (self-fooling) enabler of parental functioning; 4) continued substance use impacting parental functioning; and 5) dysfunctional parental functioning and its relational impact upon offspring.

**Discussion:** Findings suggest disruptive attachment can increase the vulnerability for SUDs on the one hand, but can be an expression of underlying trauma on the other, hence serving as a covert mechanism by which trauma can be transmitted across generations. Results indicate the need for preventive, attachment-based and trauma-sensitive interventions targeted at disruptive intergenerational patterns.

## Introduction

Mental health problems are a common co-occurring condition in substance using populations (1), with growing research acknowledging the prevalence of post-traumatic stress disorders (PTSD) in individuals with substance use disorders (SUDs) (2–6). This resulted in increased attention for integrated treatment to reduce both SUDs and PTSD symptoms (7–11). Some persons who have experienced a shocking or dangerous life event develop PTSD (12), characterized by the persistence of intense reactions to reminders of the traumatic event, altered mood, a sense of imminent threat, disturbed sleep, and hypervigilance (13). SUDs and its consequences can make individuals prone to experience stressors or trauma, which may contribute to the development of PTSD, just as PTSD symptoms have been associated with substance use initiation.

### Interpersonal Trauma and Attachment

Traumatic experiences that occur early in life within attachment relationships, often referred to as “interpersonal trauma”, are known as a significant predictor of SUDs in later life (14–17). Childhood experiences of being physically or emotionally abused or neglected or sexually abused by a trusted caregiver increase the likelihood that an individual will develop SUDs (18, 19), and have been linked to patterns of insecure attachment in offspring *via* symptoms of depression (20). Exposure to trauma within the caregiving system is associated with higher levels of affective/physiological, attentional/behavioral, and self/relational dysregulation in addition to post-traumatic symptoms (21), affecting individuals’ capacities for emotional understanding and processing significantly (17). The caregiving system may include biological parents or other relatives (e.g., foster or adoptive parents), staff in residential child care, or even therapists (22). Exposure to multiple and chronic interpersonal trauma experiences in the relationship with caregivers, also known as “developmental trauma” (23), is associated with a complex range of symptoms and impairments across several areas of development (21). Early trauma experiences with caregivers have repeatedly shown to have a profound influence on physical (24) and mental health (25), with enhanced risk for psychopathology, including dissociation and hallucinations (26). Hence, the absence of secure attachment relations is likely to result in poorer child outcomes, with trauma perpetrated by caregivers further amplifying these poor outcomes (23). Dugal and colleagues (27) indicate how dysfunctional early interpersonal encounters may shape dysfunctional interaction patterns to be repeated in subsequent relationships. They further state that, in reaction to the perception that the other is insufficiently available to answer one’s own needs of love and protection, interpersonal trauma survivors generally present a hyperactivation of the attachment system. This can be represented by intense demands of affection, a sensitivity to perceived or real threats of rejection by a partner, a certain control over a partner’s behavior, or an excessive dependence toward a partner (27). Individuals exposed to early interpersonal trauma also show an atypical combination of anxious and avoidant attachment styles that often result in severe affect dysregulation and psychopathology (17).

According to Bowlby’s attachment theory (1982), babies have a primary need to establish an emotional bond with a caregiving adult from birth (28), characterized by the need to seek and maintain proximity to a person (29), especially when the baby or child is faced with internal or external stressors (30). As such, an “internal working model” (31) is developed by the unique patterns of infant behavioral responses to primary caregivers (32), containing complex mental representations of the self, the caregiver and the quality of the relationship (33), functioning as a mediator of attachment experiences (34). These representations tend to be extended into adulthood (17). In certain cases, parents are not able to provide a safe haven for their children, offering them frightening or unpredictable caregiving (27). As a consequence, experiences of interpersonal trauma can be detrimental to the core conceptual system (35) and can become permanently imprinted in an individual’s internal working model (31), including ensuing long-lasting effects on attachment and interpersonal relationships in later life. Early attachment relationships are not always sufficiently positive to cultivate a sense of security in a child’s world (17). Moreover, insecure attachment can serve as a vulnerability factor for alcohol (36) and other SUDs (37, 38) and may contribute to early drop-out in treatment, whereas secure attachment bonds provide a sense of safety, comfort and predictability for individuals with SUDs (39).

### Mothering and Substance Use

A growing body of research reveals a high prevalence of interpersonal trauma (40–42) and insecure attachment (43, 44) in women with SUDs. In particular in mothers with SUDs, anxious-insecure attachment patterns (45, 46) may lead to difficulties when interacting with their children (e.g., inconsistency in the ability to perceive and respond to babies’ signals) (47). Especially prenatal substance use has been demonstrated to be a major public health concern affecting children (48), since chronic in utero exposure to licit and illicit drugs is associated with adverse fetal, neonatal, and early childhood consequences (49). Also perinatal substance use tends to have a detrimental effect on mother-child bonding, as illustrated in many studies (50, 51). Maternal substance use is further associated with psychiatric comorbidity (52), maladaptive parenting practices (53, 54), emotional unavailability and uncertain reflective functioning (55, 56), a lack of mentalizing abilities (57), and poor infant development (58, 59), including disruptive attachment patterns in children. A recent brain imaging study (60) revealed that mothers with SUDs showed reduced activation in key reward regions of the brain in response to their infant’s cues. Even when drug use is discontinued/controlled, psychological and relational dynamics underlying the development of parenting may be affected, limiting reflective parental functioning and challenging the quality of the parent–child relationship (57). Exposure to parental substance use eventually increases the risk of SUDs (61, 62) and other mental health problems in offspring (63, 64), which might install an intergenerational cycle of psychopathology.

Research has shown that a lack of consistent and responsive parenting is thought to interfere with the development of secure attachment in children (65). Attachment can be understood as providing a context in which we learn to make sense of ourselves and others (66). Infants are entirely dependent on their caregiving environment for safety and nurturance and their experiences within this environment are key to their developmental trajectories and longer-term outcomes (67). Emotional security, based on emotional bond between a child and caregiver, depends on the availability and responsiveness of the primary attachment figure, usually the mother (68). Attachment research suggests that the ability to regulate distress and to cope with negative feelings in relationships is learnt through attachment. Fearfully attached persons have not acquired sufficient affect regulation strategies, and can easily become anxious in interpersonal (attachment) relationships (69). In this view, maternal insensitivity and unresponsiveness to children’s emotional cues can be seen as a function of the caregiver’s own unmet attachment needs, stemming from the caregiver’s own experiences with early caregivers. Abundant research has focused on interventions for promoting the quality of mother–child interactions in combination with drug treatment (70, 71). The relationship between specific individual and relational factors can explain the fragile parenting capacities of parents with SUDs, apart from substance use (57). Treating drug addiction and promoting secure attachment bonds are increasingly recognized as two essential components of present-day mental health care (72).

Maternal substance use can impact child development adversely, since it not only increases the risk for disruptive attachment patterns (73), but also for SUDs in offspring. Adverse childhood experiences, including growing up in a context of maternal substance use (74), predicts an earlier age of onset for alcohol (62, 75) and other drug use (76, 77), and increased odds for attempting suicide (78). A positive relationship was found between maternal substance use and the occurrence of child maltreatment, indicating a clear link with insecure attachment in children and adults (65). A high incidence of emotional and physical neglect is documented among substance using mothers (57, 79, 80), as well as a greater tendency towards depression and more chaotic child-rearing environments (81). Consequently, parenting interventions need to be provided to women in substance abuse treatment, focusing on increasing maternal sensitivity, reducing harshness and providing children with sufficiently stimulating environments (82).

### Childhood Trauma and Impact on Parenting

Caregivers who have been exposed to trauma face various challenges when providing sensitive, responsive and nurturing care to their young children (83). Trauma exposure is associated with greater parenting distress and increased risk for dysfunctional parent–child relationships (84). A systematic review by Christie and colleagues (85) provided evidence for the association between parental PTSD and impaired functioning across a number of parenting domains, including increased levels of parenting stress, parenting satisfaction, and suboptimal parent–child relationships. Childhood trauma may affect the formation of early relationships and corresponding defense mechanisms in adulthood (86). Parents with a history of childhood adversity are at risk of developing problematic parenting behavior in relation to their offspring, including child abuse and neglect (87–89). Cross and colleagues (90) found that trauma in parents may impact parental distress and the risk of child abuse, potentially increasing the risk of trauma symptoms in offspring. Women who reported childhood trauma and substance use and co-occurring disorders are at increased risk of developing an intergenerational cycle of abuse (91). Finally, addiction may lead to disruption of the chemical balance critical for self-awareness and self-control (92), which can indirectly predispose a mother to abuse or neglect her child(ren). However, suchlike discourses can contribute to a negative view regarding substance using mothers’ identity as being restricted to that of a drug user, hindering the construction of new roles, such as being a mother (93).

### Aims of This Study

Despite awareness on the long-term consequences of maternal substance use for child development, research on parenting experiences from the viewpoint of mothers with a history of substance use is scarce. Although interpersonal trauma was initially not the focus of this study, mothers’ parenting experiences were clearly affected by childhood trauma experiences. Consequently, this study provides a deeper understanding on how a history of interpersonal childhood trauma may affect parenting beliefs, attitudes, and behaviors among mothers with SUDs and illustrates how trauma can ultimately persist across generations. In this study, mothers who misused illicit substances when upbringing a child reflect on their parenting experiences, based on retrospective accounts of their own childhood and their offspring’s early upbringing. Improved understanding of intergenerational trauma transmission in substance using mothers and their children would be an important step toward supporting women at risk of developing substance use disorders arising from traumatic childhood experiences. This study will thereby try to add knowledge on the emerging literature exploring potential applications of attachment-theory informed interventions in individuals with SUDs (94), especially in mothers.

## Materials and Methods

### Study Design and Procedure

A qualitative research design was applied, using in-depth interviews (one-on-one) as method for data collection. As focusing on lived experiences of individuals thought to be vulnerable or marginalized is relatively new (95), the objective was to provide an improved understanding of lived parenting experiences among women with SUDs by making new, significant distinctions resulting from getting closer to this phenomenon (96).

Inclusion criteria for participation in the study were: i) being a mother; ii) having a history of problematic illicit substance use during the upbringing of a minor child (older than 1 year); and iii) being in substance abuse treatment for at least one month. Individuals who only used alcohol or only used illicit substances before or during pregnancy were excluded. Abstinent individuals as well as individuals who were still using drugs despite being in treatment were included, given their alternative and complementary points of view. Participants recruited in inpatient treatment services were all abstinent since admission or for a longer period. Participants recruited through outpatient treatment services commonly used in a controlled way, except for two women who were abstinent. The average time in treatment for inpatient participants (n = 11) was 3.8 months (range 0.5 to 12 months), while mean treatment duration among outpatient participants (n = 12) was 4.2 years (range 6 months to 11 years). Participants’ mental state at the time of the interview (i.e., if they were emotionally able to participate) was considered with treatment providers. The accuracy of participants’ answers was openly discussed during three interviews, which led to more consistent and accurate responses.

In total, 23 mothers were included in the study (cf. **Table 1**). The average age of participants was 34 years (range 25 to 49 years). Most women had only one child. Participants’ children were between 1 and 21 years. Most mothers were involved with child protection services, including 15 mothers who lost custody of their child by court order (e.g., out-of-home placements in child care services or foster care) during the upbringing. While polysubstance use was commonplace among study participants, eight women used amphetamines when upbringing their children while nine mothers reported problematic alcohol use at that time. Detailed information regarding the characteristics of study participants is described in **Table 1**.

**Table 1:** Participants’ characteristics (n = 23).

Characteristic	Value	n = 23
**Ethnicity**	Western European South Slavic	22 1
**Relationship status**	Single or divorced Cohabitating Incarcerated partner	14 8 1
**Number of children**	1 2 3 5	11 6 5 1
**Primary substance of choice at the time of upbringing**	Amphetamine Cannabis Cocaine Heroin GHB	8 6 5 3 1
**Current substance use status**	Clean Active use	13 10
**Treatment modality**	**Inpatient treatment programs** Medical detoxification Short-term residential treatment^1^ Long-term residential treatment^2^ **Outpatient treatment programs** Substitution treatment (e.g. methadone maintenance) + individual counseling Pharmacological based treatment and/or individual counseling^3^	**11** 2 3 6 **12** 2 10
**Number of mothers who lost custody of their children**	Voluntarily assisted by child protection services Court-ordered measure	4 15

^1^group therapy, lengths of stay between 4 and 6 weeks.

^2^group therapy, lengths of stay between 6 and 12 months.

^3^twice-weekly or on a monthly basis, often with breaks in between.

### Data Collection

Interviews took place between October 2018 and March 2019. Participants were recruited from three inpatient and four outpatient substance abuse treatment services in Flanders, Belgium. Interviews were carried out at the center where participants were treated. Participation in the study was completely voluntary. Both oral and written informed consent were obtained from all participants. Prior to the interview, we explained the aim of the study and guaranteed the anonymous nature of participation. Any records that could identify the participant were made unidentifiable. Participants were free to withdraw from the study at any time, without affecting them negatively in any way. The contact details of the researcher were included in the informed consent form and participants were informed about the possibility of a follow-up conversation at the treatment center and/or with the researcher. The average duration of the interviews was 2 h and 6 min (range 00:59 h to 03:04 h). Two of the interviews were interrupted due to external circumstances (i.e., transport reasons and collecting a child from kindergarten) and were resumed a few days later. All interviews were conducted by the first author, audio-taped with participants’ permission and transcribed verbatim, after which the recordings were deleted. Transcripts were de-identified to ensure confidentiality.

A semi-structured interview guide was used in order to discuss a broad variety of themes, while at the same time asking everyone the same key questions. The guide contained questions regarding experiences with i) mothering and substance use; ii) relationship to the own parents as a child; iii) treatment and support needs; and iv) child protection interventions. In this paper, we primarily focus on data regarding the first two areas. As a reflexive and collaborative practice, we adopted a timeline approach (97) throughout the interviews, which enabled participants to talk about potentially burdening experiences from a distant viewpoint. As such, the timeline served as a tangible, mediating object between the interviewees and the researcher, giving participants more agency regarding the way they wanted to talk about their own lives and potentially harmful experiences. It enabled them to talk about adverse periods (e.g., participants pointed at a certain period, elucidating “during *that period*”), or about abusive or violent relationships, without having to identify names of relatives which could potentially evoke feelings of distress (e.g., participants referred to the timeline by constructing stories they were still suffering from: “*that one* who repeatedly abused me”).

### Data Analysis

Collected data were analyzed using a thematic analysis technique in order to attempt to describe participants’ lived experiences. In view of the scarcity of research about the experiences of these often stigmatized women, an inductive analysis was used to derive themes that popped up from the data. The process of data transcription was done by the first author, which contributed to jumpstarting the other steps of the data analysis process (98). The thematic analysis was subjected to an ongoing iterative process, following the six steps suggested by Braun and Clarke (99, 100). Initial codes were generated to identify similarities and differences in the data, which were then sorted into broader themes, with similar codes placed under the same theme. Several samples of the analysis were discussed and cross-analyzed by the research team.

### Ethics

Ethical approval for the study was obtained from the Ethics Committee of the Faculty of Psychology and Educational Sciences at Ghent University (E.C. decision: 2018/42) before commencing the project. Being aware of the vulnerable situation of participants, interviews were conducted in a trauma-sensitive way. Before and during the interview, participants’ right to declare that they did not like to talk about a specific topic without affecting them negatively, was frequently emphasized. Each interview was preceded by a brief attunement with a psychotherapist at the treatment center about the current emotional capacity of participants. Taking this information into account, the main aim at the beginning of the interview was to establish a trustful relationship, characterized by equality, trustworthiness, and safety within a secure environment. The researcher then specified the objective to gain insight into women’s lived experiences as a mother in the first place, alongside recognizing their courage to participate in the study. Throughout the interviews, the researcher tried not to be intrusive by letting trauma-related disclosures exist in its verbal or tacit constellation, always validating disclosures, and creating space for dormant silence and suffering. The researcher thereby tried to verify (in-)directly whether the participant was still comfortable with talking about the given subject, without encouraging them in any way to open themselves. Following participants’ individual pace, moving slowly, working with humility, patience, engagement, and an active listening attitude appeared to be of great importance throughout this project. Each interview was followed by short feedback to the psychotherapist on how the participant seemed to have experienced the interview emotionally, without disclosing any interview information.

## Results

**Figure 1:**
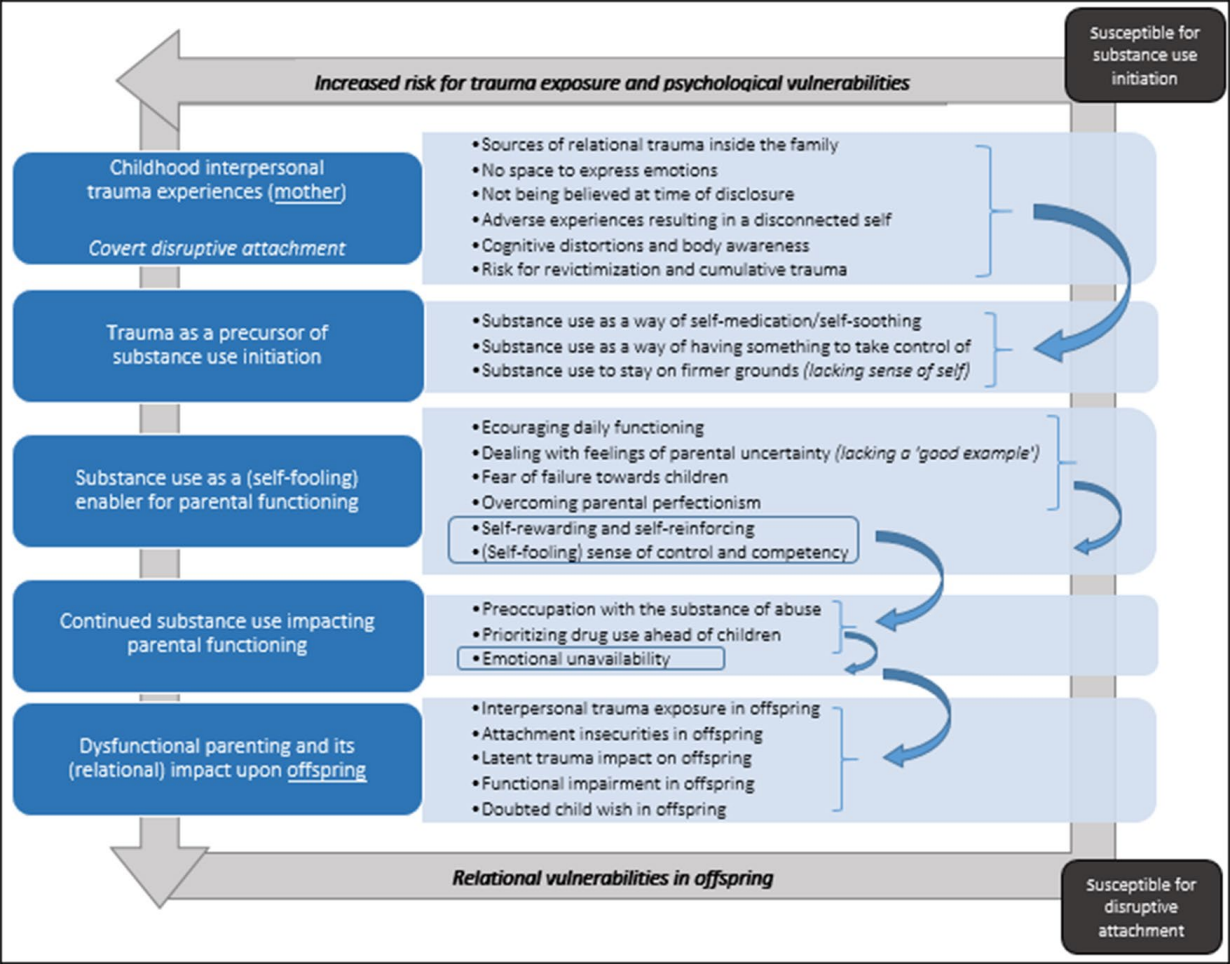
Mechanisms underlying the cycle of intergenerational trauma transmission in mothers with SUDs through latent disruptive attachment. Prolonged substance abuse and emotional unavailability stemming from preoccupation with the substance of abuse can i) increase risk of trauma exposure in offspring; and ii) install relational vulnerabilities in next generations, making them more prone to develop attachment difficulties. As such, a heightened risk for trauma exposure can be transmitted through disruptive attachment across generations.

Although not actively questioned, the vast majority of participants (n = 16) disclosed one or more interpersonal trauma-related experience(s) during childhood. Subsequently, the analysis showed an intricate link between early interpersonal trauma, attachment and addiction in the parenting narratives of mothers with SUDs, indicating the interrelatedness of these concepts. Five key mechanisms behind intergenerational trauma transmission were identified that contribute to disruptive attachment processes: 1) early interpersonal trauma experiences, 2) trauma as a precursor of substance use, 3) substance use as a (self-fooling) enabler of parental functioning, 4) continued substance use impacting parental functioning, and 5) dysfunctional parental functioning and its relational impact upon offspring. These themes and related sub-themes are represented in **Figure 1** and outlined below. The themes are presented in a presumed chronological order, as these were prominent ‘stages’, building further upon each other. However, these themes should not be interpreted linearly, and therefore rather be regarded as coexisting parts of the complex lived experiences of these mothers. By reporting on the results, specification of participants’ treatment settings are abbreviated as: OT (outpatient treatment service), IT (inpatient treatment service), and ITMC (inpatient mother-child treatment service). All names throughout the results section are pseudonyms in order to ensure confidentiality.

### Early Interpersonal Trauma Experiences

#### Sources of Trauma Within the Caregiving Environment

Speaking about parenting experiences yielded troublesome emotions in almost all participants, often reflecting upon and stemming from their own dysfunctional relationships with early caregivers. Mothers commonly experienced longstanding histories of different forms of — often repeated — sexual, physical, and/or emotional abuse, mostly inside the family. Throughout participants’ narratives, it appeared that their own parents had often gone through similar experiences as a child, not always knowing exactly what had occurred. Complex parent–child relationships and complicated and prolonged histories of abuse emerged, leading to self-destructive behavior in some participants.

“I have been sexually abused by my grandfather for years … It ultimately came out when I was in secondary school. (…) When I was a minor, I also used to harm myself … but at the same time I was experimenting with drugs. (…) When it all came out, many victims have had come to light, also my own mother … and that’s where the dispute had started. She had experienced it herself, why did she sent me to him? And if she now says to me: ‘How could you do it, taking drugs while being a mother for your kids?’, then I’m like: ‘you know only too well how that comes’. I really have difficulties coping with it. If she then wants to take over [the upbringing], then I’m really angry with her.” (Olivia, ITMC)

The realities of mothers’ lives include high rates of interpersonal abuse and violence during childhood, which often still live through. Suicidal ideation ignited by interpersonal — often cumulative — trauma softly surfaced, both directly (suicide attempts) and indirectly (suicidal ideation and/or attempts among family members, often witnessed by participants).

“In 2012, my first suicide attempt occurred. (…) My biological family has contributed to increased feelings of loneliness and unacceptance. They always said that I was stupid … everything you could blame a child for. I got locked in the basement, I got beaten. Every 5 minutes to be locked up is too much for a child, even if it’s just a minute. The world is collapsing for that child. (…) It was mainly my aunt that abused me, physically and emotionally. It was in [foreign city], we used to live there for a while. And then you arrive in Belgium, a refugee center, and there you got raped by a stranger. Those are all memories flashing back in my mind just like a movie. (…) My aunt used to drink a lot. We were with 3 children, of which 2 were hers, and if she had drunk too much, she vented her frustrations on me. I don’t know why she had to vent it on me and not on her own children.” (Charlotte, OT)

Some of the participants expressed early experiences of witnessing or finding a family member that had attempted suicide, leaving them behind with unresolved or guilt-feelings until today.

“My father has tried several times to hang up himself. Once, I found him laying down at the toilet. He had been drinking too much and taken too many pills. (…) I know he also had a tough past. His mother wasn’t very … she wasn’t such a lovely mother, she rather was a mother of corporal punishments, even though that was common practice back then.” (Evelyne, OT)

The role of participants’ parents was often reflected upon, noting several deficits in their parental functioning which goes back to the way they were raised by their own parents (participants’ grandparents). The way participants reflect on their children’s upbringing was often prompted by these early experiences and they frequently stated they wanted “to do it completely different”. One of the participants was exposed to prolonged physical abuse by her father. She had lacked a safe and nurturing environment as a kid as she grew up in a context of parental substance use herself, just like nine other participants did. From her children’s birth, she experienced difficulties in mother–child attachment and is aware that the negative experiences with her parents have an impact on her own parenting behavior. The quotation below illustrates how a history of trauma can impair a mother’s ability to accurately respond to her own children’s cues.

“I try to not transmit it to my children, however though … I’m already projecting my own experience of getting raised too strictly towards my daughters. (…) The experience of having received no love … Though I say every day to my children ‘I love you’, but it’s like … I don’t know. It’s another kind of love, you see? Like, if children got born, a mother will be crying. I didn’t with neither of the kids. I don’t know why, but I just didn’t. But I always cared for my children when they were young. (…) The way my parents behaved towards me, I already tend to transmit it towards my children, and I don’t want that. So I already started working on it now.” (Aubree, IT)

#### No Space to Express Emotions Within the Family Environment

Several participants stated to have developed a ‘holding back’ reflex and attitude concerning their affective expressions, since they have never experienced space to give vent to emotions. Some declare to have been spanked as a way of ‘obeying’, which hampered their affect-regulation. As participants commonly missed a warm and nurturing environment during childhood, talking about emotions or expressing feelings was perceived taboo or deemed prohibited.

“As a child, I didn’t get the support I needed from my parents. At home, they always said: ‘You shouldn’t cry’, and ‘you shouldn’t be angry’. Yes, you may cry, but you shouldn’t be tearing the place apart. I am re-educating my parents, in view of the moment they will look after my children … Basically, you can’t control feelings. You can control your thoughts and your behavior, that’s something completely different. But you should never condemn feelings, and at home they’ve always used to be condemned, instead of the behavior.” (Evelyne, OT)

In consideration of the developmental impact of having no space to express emotions or not having received much love at home, participants try to raise their children differently.

“I find it very important that you can show your child you can have a crisis, that things can persist, but that you can rebuild yourself and recover. At home, those things have gotten shielded always. (…) Just like with my father’s suicide attempts. ‘He has it again’, that’s just what they used to say, as opposed to me, I’m open and honest towards my children.” (Chelsea, OT)

Besides the lack of emotional space, participants also declared the painful shortage of opportunities to talk about trauma-related experiences. When participants disclosed as a child, it used to be refuted as ‘invalid’. One woman, who also experienced early emotional abuse and neglect, describes the significant impact of how an attempt to disclose rape was met with disbelief, ultimately supporting her to attempt suicide.

“My parents didn’t believe me. It has hugely impacted me. (…) On the journey from school, I have been dragged off my bike by three men. I have been raped, repulsed … When I came home, I said it to my parents. My father spanked me because my bike was broken. They didn’t believe me … what … was very hard for me … My life was such a hell already, and then that. I also started using drugs at the age of 13, shortly after this happened. I never remembered faces, but I do remember voices and hands. And when I was clean, it came up very fiercely, but when I took drugs, it didn’t. It has determined a lot of my youth.” (Hazel, ITMC)

Affirmation that an account is believed and validated is seen as very important by the respondents. In addition, the fear of not being taken seriously – as a mother who genuinely loves her child despite her addiction – was ubiquitous among all participants. They felt like they continuously had to prove the love for their children because of their addiction, although ‘good mothering and addiction have nothing to do with each other’, according to the mothers.

“Once, I asked my psychiatrist: ‘Everything that I’m telling you, do you believe me?’. I received a very pedagogical answer: ‘It is my job’. I didn’t know what to say. Last week, he made me a comment, when I told him about the [adoption] ruling that it had to appear in court. He said: ‘Maybe it’s a good thing that your child will be adopted’. I said: ‘I don’t think you know what you’re talking about’. He said: ‘I do … I have children too’. I said: ‘That’s why … You didn’t have to carry them … I did … I did carry him for almost 9 months … Thanks to my umbilical cord, that child was born’. He understood and he apologized.” (Charlotte, OT)

#### Interpersonal Trauma Resulting in a Disconnected Self

Childhood trauma that was not validated or disclosures that were not believed, tend to erode participants’ self-concept and ultimately yield a disconnected self over time, also resulting in altered body-awareness. Participants mentioned how they are constantly in doubt about themselves, feeling worthless and unwanted, not feeling loved and appreciated, which can ultimately push them to drug-taking behavior. These cognitive distortions might serve as a starting point for how they perceive and relate to oneself, others and the world; since these experiences instigate self-loathing and ways of disrespecting oneself, not knowing who they really are.

“I only was able to do the things I was sure of … that I was a ‘mother’, that I was a ‘daughter from’, but who was I myself? I had no idea…” (Harber, ITMC)

“It’s more in my head, with the abuse … he totally damaged me … I do not love myself, that I am sure of. Neither do I have self-respect. These are all of the things I have to learn, but it’s difficult. As long as you do not love yourself, you do not love somebody else … and for me, it doesn’t seem to work yet … It [trauma] is still too deeply-rooted within myself.” (Aubree, IT)

#### Risk of Revictimization and Cumulative Trauma

Participants’ stories depicted an increased vulnerability for ‘looking for love and affirmation in the wrong places’, with many mothers encountering repeated interpersonal, cumulative trauma, even throughout adult life. Participants also revealed how prolonged emotional abuse tended to go unnoticed. Following quote illustrates how a cumulative interpersonal trauma history completely absorbed one mother, eventually leading to suicidal ideation.

“Before I started treatment here, I attempted suicide. And the feeling hasn’t really gone yet. I have incorporated it somewhere, but it’s still torturing me. I still have the feeling my backpack is almost full. Just a little more might be needed until I would do it again. I was astonished that I had done that. You always hear that people who really want to commit suicide will never talk about it, and I can see, now that I have wanted to do this … Indeed, you don’t talk about it.” (Olivia, ITMC)

### Trauma as a Precursor of Substance Use

#### Self-Medication/Self-Soothing

All participants who experienced childhood trauma mentioned that they initiated substance use in order to self-medicate painful experiences. For example, mothers revealed how a history of broken attachment relationships and traumatic experiences played a role in substance use onset and the transition to addiction. The effects of substances enable(d) mothers – albeit temporarily – to suppress negative memories, cope with difficult emotions, and – finally – find inner peace. Without drugs, they state they would not have been able to overcome certain incidents.

“I just ‘used’ the pain away, the psychological pain, not the physical pain. A bruise will fade, but what it does to you, doesn’t. So I heavily ‘used’ it away. That’s why my drug usage had increased considerably in recent years. (….) My father was a professional soldier, he vented his frustrations on me. It became unbearable in the end, I had one bone fracture after the other. (…) My cousin has committed suicide with medication and alcohol, and I have found him … horrible … horrible to find someone that way. (…) Then drugs was easy, you shouldn’t think, you shouldn’t feel.” (Hazel, ITMC)

#### Regaining Control and Standing on Firmer Grounds

After a history of being controlled, mothers perceived substance use as something they could control and have power over again. At the same time, *participants’ own choice to use substances* instead of looking for help elicited narratives of self-blame.

“I used to do athletics on an international level. I was very good. My parents have never motivated me to continue doing what I was good at and loved to do. That made me think that it [drugs] was really something that belonged to me, that could comfort me and that I chose to do myself. I chose to use drugs myself.” (Charlotte, OT)

Participants also asserted how substance use enabled them to stand up for themselves again.

“Because of the drug, you just dare to do more. ‘I don’t care’. You don’t do it purposely, but you dare to speak up and that goes from the store, open up your mouth to a saleswoman, or to a partner.” (Noelle, OT)

### Substance Use as a (Self-Fooling) Enabler of Parental Functioning

#### Enabling Daily Functioning

Participants described various ways in which they perceived that substance use enabled them to manage their parenting roles (e.g., doing the household), which is illustrated by the quotation below about a mother using amphetamines in order to be alert enough to function during the day and to ensure that she could attend to her child’s needs.

“Speed helps me a lot, but I don’t abuse it. I take just as much in order to calm down and focus, so that I can do what I need in order to go through the day. (…) It’s on a daily basis, but I’m working. I live in my home, my kid is nicely dressed, I’m taking him every day to school, to the speech therapist, everything … Without speed, I wasn’t gonna make it. I wasn’t gonna have the energy to do what I am doing. It just helps me through.” (Elliana, OT)

#### Dealing With Feelings of Uncertainty

Mothers stated that they used substances to deal with feelings of uncertainty and fear of failure as a parent, seemingly stemming from unresolved trauma in the past, along with lack of good examples in their own environment. Some mothers were afraid that their children would get short-changed, not receiving the affective attention they need.

“I played board games with the kids, I went for a swim with the kids, we went to a theme park. I just did it all, maybe a little too much. For me, those were nice moments, but I am afraid that, if one day I will come home and there will be no speed, that these things might not happen anymore. I am afraid of becoming clean … that I will disappoint the kids: ‘mom, before, you used to be like that, before, you used to do that with us’… I am afraid actually that I will be a deficient parent.” (Whitney, IT)

#### Self-Rewarding and Self-Reinforcing Mechanisms

Some mothers mentioned that substance use was a means of self-reward (for example at the end of the day when children were asleep) and slowly became a self-reinforcing activity.

“Sometimes I also led a double life. Taking drugs at night, and in the morning you have to be there as a mother, which has taken its toll. You are tired, irritable. You are using up your reserves. So you start using again in order to get new energy from your body, which brings you in a vicious circle and you get tired even more. So you use more and more, until you become over-tired, not functioning well so you have to sleep.” (Elizabeth, ITMC)

Alongside with self-reward, however, came feelings of guilt, followed by a search for affirmation from their children.

“I always made sure she had everything she needed: her food, her bed, everything in time. However, on an emotional level, you know it yourself, you’re not yourself. Your child notices it, feelings of guilt arose, and from the moment she was laying in her bed, I used to consume … But from the moment I realized, I felt guilty and bad.” (Noelle, OT)

#### Self-Fooling Sense of Control

When reflecting on the upbringing of their children, mothers in inpatient treatment agreed that they are now able to challenge the ‘false narratives’ they had developed. While experiences of interpersonal trauma had resulted in feelings of inadequacy, substance use and its effects equipped them with an apparent sense of being good enough and competent as a mother, ultimately leading to a self-fooling sense of control.

“I kept going, also for my children. I’m not saying I have done well, but … not giving up. Also taking care of his [ex-partner] children, but always in combination with substance use but trying to hide it as much as possible for the children, for the outside world. Trying to linger in family life in a good way, at least in my head. (…) In the morning, when the kids woke up, I opened the curtains, their sandwiches were spread, … Anyway, I thought I got it all, but that was a very false sense of ‘I’ve got everything under control’ and that feeling has held me back for so long.” (Sofia, IT)

Respondents mentioned that motherhood is often characterized by a lack of self- and parental confidence and, consequently, fear of failure. While substance use may be perceived, particularly by amphetamine users, to help them to overcome these issues and enable parental functioning in the short-term, these benefits appeared to be transient.

“I think that everyone who is still using drugs, is going to say: ‘yes, I can raise a kid while using’. But I think it isn’t. I have used for 32 years, 32 years I have been thinking: ‘I can handle the whole world’, but in fact, I have ruined so much, instead of dealing with things and searching for help and not taking drugs … Problems exacerbated and I only used more to push them away … Using drugs doesn’t make you more human. You’re a little doll, a marionette, doing what’s expected, but who’s not feeling and thinking.” (Hazel, ITMC)

### Continued Substance Use Impacting Parental Functioning

While substance use may help some mothers to avoid re-experiencing trauma, it gradually progressed to losing control over substances in many mothers. The following quote illustrates a mother’s recognition of how prolonged substance use hindered her functioning as a parent:

“I still wanted to do it all, because of the speed. I sat down in the seat to watch tv with her. So I pushed myself to still give her everything, even though she will have had the feeling: ‘Mom isn’t calm’. A child feels that, but in my world, I tried to do my best, but at the end, it just didn’t work anymore.” (Abby, OT)

#### Preoccupation With the Substance

Parenting was hampered through mothers’ preoccupation with substances of abuse, while everything else, including their own children, was secondary. Mainly women in residential settings described how it is impossible to maintain an addiction together with child care, as they would do anything to get their drugs. This insight often went along with feelings of not having been a good enough mother.

“In the end, we are not focused on what’s happening around us. I’m going to be very rude, but if you have almost nothing left, you’re constantly busy with: ‘how should I get the drugs now?’ In the end, it’s the drugs that you live for and not your kids … Now that I’m clean, I realize.” (Olivia, ITMC)

#### Prioritizing Drug Use Above Children

Preoccupation with the substance ultimately resulted in prioritization of drugs above the children. In addition, participants mentioned that, when being under the influence, children’s needs were perceived as being ‘too much’, more than they could handle at the time. Some mothers unravelled profound and honest stories in light of prioritizing substance use and its impact on parenting.

“I would have gone through a wall for it [cocaine]. When I had used, I could keep myself busy for 6h with only one pill. Nobody would have talked to me, I was very focused, living in another world, neglecting everything, neglecting my kids. Sometimes I used in presence of them. I was in my own world, ignoring everything. They had to leave me alone. I put off my bell, my phone, I locked my door, everything … so I didn’t had to talk to anyone. I just wasn’t able. I was just busy with that [drugs], and only that.” (Aubree, IT)

#### Emotional Unavailability

After some months in treatment, mothers looked back and recognized that they had been unable to respond to their children’s emotional needs. Although physically present, they admit that they have done considerable harm towards their children and that they were emotionally absent.

“At a given moment, my usage was very problematic. I used almost on a daily basis, and it did impact the upbringing. If I think about it now when I am sober … At home, everything was always fine, it was clean, I used to clean a lot. The kids had fresh clothes every day, they had everything they needed, no shortcomings. They were fine with everything, but that’s it. I noticed it was very hard for me to stay focused on the kids, to play with them, to talk with them … I was more introvert … But now [clean], now I have this relational thing with the kids. I am really occupied with the kids. I play with the kids.” (Olivia, ITMC)

Participants frequently referred to their unavailability despite their physical presence. Participants described themselves as unable to express warmth and affection and to interact appropriately with their children. Narratives of being a good mother ‘under the influence’ tended to be counterbalanced by the insight that, in fact, they had forsaken their parenting role in its entirety. Consequently, drugs can be seen as an important mediator of trauma throughout the stories, as mothers are less attentive, accompanied by a risk of transmitting trauma.

### Dysfunctional Parental Functioning and Its Relational Impact Upon Offspring

#### Offspring Exposed to Interpersonal Trauma

As a consequence of maternal substance use, participants’ stories revealed how their children had been exposed to things they should not have been exposed to, both at the hands of their caregivers and as a lack of maternal involvement and alertness due to substance use, so say the respondents.

“I have neglected my daughter a lot. I haven’t given her a lot of love. I left her behind everywhere. You don’t want to know it … That child has seen too much. That child is still suffering from it.” (Aubree, IT)

Respondents’ children were confronted with vigorous life stressors, already from a very young age and sometimes even prior to birth. Violence committed by a former partner towards the children as well as towards the mothers was not an isolated case.

“My ex has tried to strangle me in presence of my child … [He] locked me up underneath the stairs in presence of my child.” (Evelyne, OT)

“I was literally waiting for him to kill me … because sometimes, he grabbed my throat and squeezed it very hard. I used to see it myself a lot with my mother, we had to flee ourselves, literally with a teddy bear in our arms, we ended up there in a shelter. So, no, I don’t want the kids to experience that.” (Whitney, IT)

#### Resulting in Insecure Attachment in Offspring

The excerpt below demonstrates how mothers’ childhood experiences – and subsequent substance use – are linked to insecure attachment in their children.

“I notice it now with my son also. The things he wrestles with [separation anxiety], I am partially responsible for it. If I wouldn’t have abused drugs, it might not have turned out this way.” (Hazel, ITMC)

The following quote shows how a mother’s past experience of sexual abuse is still alive in some form of discomfort, when being hold by her daughter. As a consequence, her daughter suffered from separation anxiety, with relational problems persisting across generations through patterns of insecure attachment.

“When my mother saw me hugging my friend, she began to cry because I had never done that at home, and it got passed on to my daughter. I can’t bear that she hugs me for more than two minutes. I’m starting to feel cramped and I’m like: ‘It’s ok, H.’. But I transmitted it to her, I’m trying my best. (…) To me, those arms, those hands, they burn through my body. And with that I’ve been … [sexually] assaulted … by my grandfather … since then it got worse, although I’ve always had that … but since then, it got worse. (…) She cannot live without me. She always used to cry when she didn’t see me, or used to panic when she didn’t find me immediately.” (Lexi, OT)

#### Latent Trauma Impact on Offspring

Respondents mentioned that feelings of anxiety and stress originated in their childhood as a consequence of frightening, threatening situations and interpersonal trauma. They stated that their children often experienced the indirect impact of mothers’ latent trauma (i.e., ‘using the past away’). While one mother blames herself for not having seen and intervened when her daughter was sexually assaulted by her partner, another woman recounts the cumulative effects of multiple suicide attempts – stemming from unbearable suffering – on her child. Mothers portrayed the troubles their children face today in light of their own complex past and present, which is often accompanied with shame, regret and feelings of having failed.

“My daughter got sexually abused by her father, and that’s also why I got depressed, because of what had happened to my daughter. I’ve been there myself, not by my father, by other men, repeatedly, unfortunately … I have never talked about it to anyone. My parents do not know. (…) Taking drugs suppressed my emotions. (…) If I didn’t take drugs, I probably might have better noticed that my man touched my daughter. Do you understand what I mean? That’s something I do realize now, now I am clean. If only I had been so smart in the past, just to cope with all of that and wanting to feel.” (Stella, ITMC)

“I have done 4 attempts … I always tried to do it with medication, except for once, when I cut my wrists. My daughter was already born then, she even saw it. Because I remember that I said to her that I had cut myself wanting to throw something into the garbage … she still remembers.” (Chelsea, OT)

Several mothers had children with a developmental disability, including attention deficit hyperactivity disorder (ADHD), autism spectrum disorder (ASD), learning disorders or attachment disorders, anxiety, etc. Latent trauma among one mother seemed to persist in offspring in terms of speech development.

“My son has seen things that were absolutely unacceptable. And he still remembers. David [ex-partner] had knocked out my teeth, my son has seen it. I also have been grievously maltreated by my last partner and he has seen a lot of it, which is not ok … and that’s because of my drug use, at such moments, I wasn’t able to protect my child. It has hugely affected him. When he started in the childcare center, he also had completely stopped talking. He only wanted to talk to me, and not to the childcare workers, which generated a speech impediment.” (Hazel, ITMC)

Ultimately, the complex lives of these mothers may have unexpected and unwanted consequences for the next generation. One mother reported about the broken dreams of her daughter, after having been exposed to intimate partner violence and missing her mother for several weeks due to a residential treatment episode.

“It still breaks my heart. A couple of days ago, Margot [daughter, 10 years] told me: ‘Basically, mom, when I bear in mind everything that you have encountered, I don’t want to have children anymore.’ This really touches my heart.” (Noelle, OT)

## Discussion

Based on 23 interviews with mothers with SUDs in inpatient and outpatient treatment, this study aimed to provide a profound understanding of parenting experiences through qualitative content analysis. The struggle among mothers to self-regulate frequently shared an etiological base of attachment system dysfunction (101) due to interpersonal childhood trauma. Given the importance of understanding the mechanisms that may promote or impede connections between mothers’ own experiences of interpersonal trauma and difficulties in fostering secure attachment with their (young) children, analyses were conducted taking these experiences as a starting point. The current study applies an attachment-theoretical framework to the association between traumatic childhood experiences and substance use and substance-related problems, demanding an alternative etiology of substance abuse — as a symptom of an unmet need that fuels an individual’s attraction to a particular substance, rather than a stand-alone disease (102, 103). From this point of view, the self-regulation framework is introduced.

### The Self-Regulation Framework: Understanding Object-Relation Re-Enactments

Findings of this study highlight that the ability to calibrate regulatory responses may be compromised among mothers with SUDs and attachment disorders, emphasizing the need to view SUDs within an attachment-based perspective (101). MacLean (104) suggested that opiate use serves as a substitute for relational attachments, which has been an impetus for a growing body of research focusing on addiction as a way of affect regulation. More specifically, addiction can be seen as a transfer by which “affectional bonds” are replaced by “addictional bonds” (105), thereby serving as an alternative means to self-regulate (101). Consequently, seeing addiction as a symptom of early relational trauma (106, 107), substance abuse can be regarded as “an attempt at self-regulation in the service of adaptation” (101), relying on something other than nurturing relationships as a way to nurture the self (108). According to Shults (108), this study reveals how negative self-perception, broken relationships with others, and a distorted world view *via* distorted perceptual filters can result from attachment difficulties. Individuals’ attachment-seeking behavior, originating from early broken bonds (109), may ultimately lead to addiction, while representing a re-enactment of past trauma as a defense strategy (110). In this view, the self-medication hypothesis maintains that suffering is at the heart of addictive behaviors (111), with the latter providing the soothing and safety which are the features of an internalized secure base (109). “In the same sense that the attachment figure is sought out when the infant experiences increasing anxiety, the drug of choice may be urgently sought as a substitutive object later in life” (112, p. 61), referring to the predictability and reliability that they might have lacked as a child.

Traumatic childhood experiences can disrupt individuals’ psychological stability (113), with attachment vulnerabilities as the root for emotional and adjustment problems, providing mothers with re-enactment desires to a drug of abuse in order to enhance feelings of security and create genuine self-esteem and self-regulatory functions (30). Moreover, lower self-regulation has been associated with SUDs (114). On the other hand, incomplete regulation (115) and less “controllability” (114) can make individuals also more susceptible for substance abuse. Hence, according to a growing body of research (116), childhood trauma can serve as a risk factor for developing SUDs. Fuchshuber and colleagues (86) also confirm that relationships of childhood trauma and personality organization can promote the understanding of individuals developing SUDs. Weegmann and Khantzian (117) describe how ordinary attachment needs and attachment to inanimate substances carry equal features, such as proximity maintenance and homeostasis.

Notwithstanding the persistent impact of insecure attachment throughout the lifespan, a growing body of research emphasizes that attachment representations are not permanent and can evolve across the lifespan (118). The protective role of sensitive caregiving is especially vital in the context of stress and trauma (83). Moreover, parent-child attachment also seems to be a major theme in protecting adolescents from substance use (119). Several psychotherapeutic interventions show promise in ameliorating the types of caregiver-child relationship difficulties that are common among trauma-exposed parents and their young children (83). Also among individuals with SUDs, traumatic attachments can be replaced by healthier, human attachments of various sorts (117, 118). Iyengar and colleagues (118) indicated that mothers with a history of trauma can transition towards secure attachment, based on their enhanced understanding of past and present experiences. Furthermore, children of substance using women can be attached securely, indicating the potential to break the cycle of insecure attachment transmission across generations (120). A recent empirical study in a group of prisoners undergoing therapeutic community treatment revealed an increase in secure attachment after one year of treatment (121). Bortolini and Piccinini (122) revealed consistency in mothers’ experiences with their own caregivers characterized by affective, sensitive care and their children’s secure attachment, indicating patterns of attachment can be securely transmitted across generations. These are encouraging findings, given the protective role of sensitive caregiving and secure attachment (83, 123, 124), and since attachment security mitigates trauma-related stress (125).

### Impact of Trauma on Parenthood and Intergenerational Trauma Transmission

According to Vanderzee and colleagues (126), our study findings confirm the high prevalence of intergenerational trauma among families impacted by maternal substance use. The finding that mothers’ disrupted bonds with their parent(s) led to a lack of trust in their own parenting capabilities, concurs with recent research elucidating impaired parental functioning in adults who grew up in a context of parental substance use (127). Participants reported to have experienced emotional abuse and neglect as a child, resulting in insecure attachment and high parental stress. The long-lasting relational effects of childhood interpersonal trauma may impede parents’ capacity (128), increasing the risk of abusive or neglectful behavior towards their own children and maintain the risk of intergenerational cycles of trauma.

Our findings suggest that not only experiencing early interpersonal trauma, but also witnessing such events can severely impact child development (19). A growing body of research focuses on the potential effects of witnessing violence in children (129). Pynoos and Nader (130) examined traumatic responses of children who witnessed sexual assault of their mothers. The researchers found that these children exhibited, amongst others, prominent PTSD-symptoms, alterations in their sense of security and vulnerability, challenged self-esteem, and stress in intra-family and peer relationships. In addition, exposure to child maltreatment (14) and exposure to stress early in life (131) have been associated with a heightened vulnerability for developing SUDs. Streeck-Fischer and van der Kolk (125) refer to the negative prospects of these children in the absence of prevention or early intervention, since they are likely to grow up and lead traumatized and traumatizing lives, as their problems with affect modulation are likely to lead to impulsive behaviour, SUDs and interpersonal violence.

Parental trauma history may intervene with the ability to foster child development (132). Findings of this study reveal how parental trauma can be transmitted through insecure attachment patterns when the mother has experienced early relational trauma. Padykula and Conklin (101) state that interpersonal trauma affects the capacity for emotional regulation negatively, because the emotional subsystem is predicated upon incongruent mirroring. Salberg (133) puts this very well: *“It is because of attachment’s primal aspect in our psyches that trauma and its impact constitute massive disruption and disorganization of the parent–child bonding system. When trauma revisits us transgenerationally through disrupted attachment patterns, it is within the child’s empathic attunement and bond that the mode of transmission can be found.”* In this respect, Brothers (134) refers to the concept of “traumatic attachment,” profoundly affecting parent–child interactions over generations. Experiences of early interpersonal trauma can affect parental functioning negatively (135), leading to adverse experiences in second generations. Parents who have traumatic histories themselves and consequential disruptive attachment styles tend to communicate these dysfunctions to their own children who may later develop disruptive attachment patterns (65).

### Clinical Implications

Mothers with experiences of early trauma and SUDs are typically fighting against distrust, nonetheless searching for a safe caregiving environment and a genuine therapeutic relationship. Findings suggest this is particularly necessary at the start of care trajectories, allowing ways to properly express feelings related to insecurities in upbringing their children as well as previous events and contemporary emotions in an unprejudiced way. Awareness of mechanisms behind parental uncertainty and intergenerational trauma transmission is needed among practitioners, as well as the recognition of attachment difficulties in mothers with SUDs as a manifestation of underlying trauma. Hence, the impact of early trauma on mothers’ parental functioning and the establishment of safe bonding in the newborn should be addressed. Coping with and healing from early trauma, validating its impact and parenting support in the critical first years of life can help mothers to stand on firmer grounds concerning their parenting capacities and to turn their pain into growth. Moreover, increased self-control, resilience, and self-esteem can enhance self-efficacy among individuals with SUDs (136).

Emphasizing the importance of different factors in the etiology, development, and maintenance of addictive behaviour (137), study findings highlight the necessity of applying attachment-enabling interventions in substance abuse treatment, to be designed and delivered in a trauma-informed manner to promote parent–child bonding and healing as a parent in the first place, as self-concept and mothering are deeply related to each other (138). Practitioners need to be aware of the enormous suffering that is at the root of SUDs (117), with a need for treatment to be based in providing what was lacking (30), mindful of the healing potential of trustful interpersonal relationships in the aftermath of trauma. In this respect, the Attachment, Self-Regulation and Competency Model (ARC-model) (22) emphasizes the importance of (re-)building safe relational systems, recognizing the core effects of trauma exposure on relational engagement, self-regulation, and developmental competencies. Furthermore, contacts with treatment providers foremost need to exhale a secure base of safety and confidentiality, where women should feel taken seriously. Consequently, results highlight the importance of a trauma-informed organizational culture in substance abuse treatment. Although there is no consensus regarding the content and modality of an integrated treatment approach focusing on trauma and SUDs (139), this study stipulates its necessity for substance abuse treatment. Our clinical recommendations are in line with Isobel and colleagues (140), who identified two contributing constructs for the prevention of intergenerational trauma transmission: “resolving parental trauma” and “actively supporting parent‐infant attachment.” Findings reveal the importance of promoting trauma-informed parenting interventions for facilitating secure emotional connections between mothers with SUDs and their children. In addition, supporting mothers in developing alternative pathways for dealing with their suffering requires that trauma disclosures should always be validated and processed within the therapeutic framework, in close consultation with mothers themselves and with their needs in this regard as a guiding principle. In addition, the high risk of re-traumatization and subsequent early drop-out among women needs to be considered. It is important to create a safe environment in which women can build on a safe sense of being and a validating sense of self. Given that practitioners are adequately supervised concerning the impact of trauma and how to carefully deal with it, trauma-related experiences should be systematically assessed within the treatment protocol (141), and — if desired by the individual — addressed. Hereby, it is of utmost importance for practitioners to be trained and clinically supervised in trauma dynamics, in order to deepen their understanding of the impact of trauma on their work culture, as well as to protect practitioners from vicarious trauma (142).

### Study Limitations

A first limitation of this study is the small number of participants, limiting the generalizability of our findings. Second, the effects of long-term therapeutic treatment on mothers’ attitudes and reflective abilities need to be considered. Mothers taking part in this study might have been better aware of their psychodynamics, attachments, traumas, and the impact substance use had on their life and children, particularly mothers engaged in mother–child residential treatment. Third, a significant number of mothers lost custody of their children. Participants were skewed towards those with involvement in the child welfare system, indicating the severity of the cases and thus not representing substance using women in general. Lastly, while our focus here was on mothers’ reports, we recognize the importance of multiple perspectives (e.g., partner, parents, children, etc.) to enrich our understanding of these transmissions’ complexities from a transactional framework, as well as multiple research methods (qualitative and quantitative). Despite these limitations and although there are additional moderators of intergenerational transmission of trauma that we did not capture within the scope of this study, it is one of the first studies that tried to give an in-depth understanding of intergenerational trauma transmission from substance using mothers’ perspectives. Insights derived from this study can be helpful for practitioners to decrease mothers’ chances of getting involved in destructive re-enactments and diminish vulnerability across generations (110). By understanding the structure of dysregulated parenting among mothers with SUDs and a history of relational trauma, clinicians will have practical information to specifically target interventions to disrupt maladaptive parenting practices and cognitions, making this research valuable for better understanding and enhancing a mother’s journey towards recovery from substance use as well as post-traumatic growth.

### Recommendations for Further Research

Given that this study as well as other research have noticed a substantial prevalence of childhood interpersonal trauma exposure in women with SUDs, and given the detrimental impact of cumulative trauma on parental functioning, further research should focus on how trauma-informed, effective parenting interventions can be integrated into substance abuse treatment. Also, between group differences (abstinent vs. non-abstinent mothers) regarding trauma transmission and attachment mechanisms may be of interest for further research. A high priority for future research is to discover protective factors by which parents overcome intergenerational patterns of disruptive attachment in the aftermath of trauma. In order to develop evidence-based practices that integrate trauma work into substance abuse treatment interventions, more research is needed on the relationship between profiles of childhood trauma (143), the use of specific substances, and parenting and attachment styles. Such research may identify distinct pathways by which early interpersonal trauma is manifested in parental functioning and offspring outcomes. An enhanced understanding of how parental trauma impacts parenting — in a way that does not further traumatize parents — alongside insights in correlates of post-traumatic growth would be an important step toward recognizing individuals at risk of developing SUDs arising from traumatic attachments. Finally, recent studies have focused on alexithymia in substance using populations (144, 144–146), which recently has also been linked to attachment (147), childhood trauma (148, 149), and suicidal ideation (150–152). Research aimed at furthering knowledge on alexithymia in women with SUDs and their offspring would be of particular interest, as the results of this study may also be explained in terms of secondary alexithymia.

### Conclusion

Mothers in this study expressed an etiological base of attachment system dysfunction due to early interpersonal trauma experiences. In an attempt to regulate the painful emotions of not feeling attached and safe in the world, maternal substance use may increase the risk of suboptimal caregiving, perpetuating the cycle of trauma and impacting the establishment of secure attachment in their children’s lives. Problematic substance abuse and related parental dysfunction can result in mechanisms by which insecure attachment and trauma are transmitted across generations. Findings indicate the need for these concepts to be regarded in the development and implementation of therapeutic interventions for mothers with SUDs, since this study underlines the need to understand SUDs as resulting initially from broken attachment relationships. Mothers with a history of interpersonal trauma are longing for a home as — being — a place of safety instead of a place of fear, creating parental expectations for themselves which can contribute to fear of parental failure and ultimately challenge parental identification across generations.

## Data Availability Statement

Datasets for this manuscript are not publicly available because of ethical reasons and confidentiality. Requests to access the datasets should be directed to FM (Florien.Meulewaeter@UGent.be). 

## Ethics Statement

The studies involving human participants were reviewed and approved by Ethics Committee of the Faculty of Psychology and Educational Sciences of Ghent University (E.C. decision: 2018/42). The patients/participants provided their oral and written informed consent to participate in this study.

## Author Contributions

FM wrote the manuscript and was responsible for general ideas. SD and WV designed the study, provided feedback on the data-analysis process, and revised the manuscript.

## Conflict of Interest

The authors declare that the research was conducted in the absence of any commercial or financial relationships that could be construed as a potential conflict of interest.

## References

[1] Dauber H Braun B Pfeiffer-Gerschel T Kraus L Pogarell O Co-occurring mental disorders in substance abuse treatment: The current health care situation in Germany. Int J Ment Health Addiction (2018) 16(1):66–80. 10.1007/s11469-017-9784-5 PMC5814539 29491767

[2] Berenz EC McNett S Paltelln K Development of comorbid PTSD and substance use disorders. Posttraumatic stress and substance use disorders (2019). 10.4324/9781315442648-2

[3] Gielen N Havermans R Tekelenburg M Jansen A Prevalence of post-traumatic stress disorder among patients with substance use disorder: it is higher than clinicians think it is. Eur J Psychotraumatol (2012) 3. 10.3402/ejpt.v3i0.17734 PMC3415609 22893849

[4] Jarnecke A Allan NP Badour CL Flanagan JC Killeen TK Back SE Substance use disorders and PTSD: Examining substance use, PTSD symptoms, and dropout following imaginal exposure. Addict Behav (2018) 90. 10.1016/j.addbeh.2018.10.020 PMC6324999 30355535

[5] Mckee SA Hilton NZ Co-occurring substance use, PTSD, and IPV victimization: Implications for female offender services. Trauma Violence Abus (2017). 10.1177/1524838017708782 29333981

[6] Reynolds M Mezey G Chapman M Wheeler M Drummond C Baldacchino A Co-morbid post-traumatic stress disorder in a substance misusing clinical population. Drug Alcohol Depend (2005) 77(3):251–8. 10.1016/j.drugalcdep.2004.08.017 15734225

[7] Flanagan JC Korte K Killeen TK Back SE Concurrent treatment of substance use and PTSD. Curr Psychiatry Rep (2016) 18(8). 10.1007/s11920-016-0709-y PMC4928573 27278509

[8] Ford JD Russo EM Mallon SD Integrating treatment of posttraumatic stress disorder and substance use disorder. J Couns Dev (2007) 85(4). 10.1002/j.1556-6678.2007.tb00616.x

[9] Pilz R Hartleb R Konrad G Reininghaus E Unterrainer HF The role of eye movement desensitization and reprocessing (EMDR) in substance use disorders: A systematic review. Fortschritte der Neurologie-Psychiatrie (2017) 85(10):584–91. 10.1055/s-0043-11833 29017196

[10] Roberts NP Roberts PA Jones N Bisson JI Psychological therapies for post-traumatic stress disorder and comorbid substance use disorder. Cochrane Database Syst Rev (2016) 4(CD010204). 10.1002/14651858.CD010204.pub2 PMC8782594 27040448

[11] Shenai N Gopalan P Glance J Integrated brief intervention for PTSD and substance use in an antepartum unit. Matern Child Health J (2019) 23(5):592–6. 10.1007/s10995-018-2686-8 30569303

[12] Freschi E Post–traumatic stress disorder. (2010). 10.1007/978-1-60327-329-9_15

[13] Shalev A Liberzon I Marmar C Post-traumatic stress disorder. N Engl J Med (2017) 376:2459–69. 10.1056/NEJMra1612499 28636846

[14] Cicchetti D Handley ED Child maltreatment and the development of substance use and disorder. Neurobiol Stress (2019) 10. 10.1016/j.ynstr.2018.100144 PMC6430405 30937350

[15] Flores PJ Addiction as an attachment disorder. Northvale, NJ: Jason Aronson (2003).

[16] Moran PB Vuchinich S Hall NK Associations between types of maltreatment and substance use during adolescence. Child Abuse Negl (2004) 28(5):565–74. 10.1016/j.chiabu.2003.12.002 15159070

[17] Schimmenti A Caretti V Clinical issues and somatic and psychiatric pathology. Alexithymia: Advances in research, theory, and clinical practice. Attachment, trauma, and alexithymia (2018) 127–41. 10.1017/9781108241595.010

[18] Afifi TO Mota N Dasiewicz P MacMillan HL Sareen J Physical punishment and mental disorders: results from a nationally representative US sample. Pediatrics (2012) 130(2):184–92. 10.1542/peds.2011-2947 22753561

[19] Covington SS Women and addiction: A trauma-informed approach. J Psychoactive Drugs (2008) Suppl. 5:377–85. 10.1080/02791072.2008.10400665 19248395

[20] Khan M Renk K Understanding the pathways between mothers’ childhood maltreatment experiences and patterns of insecure attachment with young children *via* symptoms of depression. Child Psychiatry Hum Dev (2018). 10.1007/s10578-018-0808-6 29752663

[21] Kisiel CL Fehrenbach T Torgersen E Stolbach B McClelland G Griffin G Constellations of interpersonal trauma and symptoms in child welfare: Implications for a developmental framework. J Fam Violence (2014) 29:1–14. 10.01007/s10896-013-9559-0

[22] Blaustein ME Kinniburgh KM Providing the family as a secure base for therapy with children and adolescents. In: Intervening beyond the child: The intertwining nature of attachment and trauma. (2015). Retrieved from http://www.traumacenter.org/clients/Intertwining_Nature_of_Attachment_and_Trauma.pdf.

[23] van der Kolk BA Developmental trauma disorder: Toward a rational diagnosis for children with complex trauma histories. Psychiatric Ann (2005) 35:401–8. 10.3928/00485713-20050501-06

[24] López-Martinez AE Serrano-Ibánez ER Ruiz-Párraga GT Gómez-Pérez L Ramírez-Maestre C Esteve R Physical health consequences of interpersonal trauma: A systematic review of the rol of psychological variables. Trauma Violence Abus (2018) 19(3):305–22. 10.1177/1524838016659488 27456113

[25] Lim BH Adams LA Lilly MM Self-worth as a mediator between attachment and posttraumatic stress in interpersonal trauma. J Interpers Violence (2012) 27(10):2039–61. 10.1177/0886260511431440 22328657

[26] Gómez JM Freyd JJ High betrayal child sexual abuse and hallucinations: A test of an indirect effect of dissociation. J Child Sex Abus (2017) 26(5):507–18. 10.1080/10538712.2017.1310776 28569650

[27] Dugal C Bigras N Godbout N Bélanger C Childhood interpersonal trauma and its repercussions in adulthood: An analysis of psychological and interpersonal sequelae. (2016) 10.5772/64476

[28] White K Attachment theory and the John Bowlby Memorial Lecture 2013: A short history. In: Gill R, editors. Addiction from an attachment perspective: Do broken bonds and early trauma lead to addictive behaviors?., Routledge (2018).

[29] Vondráèková P Attachment and alcohol use disorders. Adiktologie (2013) 13(1):62–70.

[30] Dallacroce P (2015). Addiction as attachment trauma, Retrieved from https://www.dallacrocemft.com/uploads/5/4/7/1/54712887/addiction_as_attachment_trauma_web.pdf.

[31] Bowlby J Attachment and loss Vol. 1 New York: Basic Books (1982).

[32] Thomson P Jaque SV Attachment, parenting, and childhood adversity. In: Creativity and the performing artist. (2017). 10.1016/B978-0-12-804051-5.00011-1

[33] Blizard RA Disorganized attachment, development of dissociated self states, and a relational approach to treatment. Journal of Trauma and Dissociation (2003) 4(3):27–50. 10.1300/J229v04n03_03

[34] Bowlby J A secure base: Parent-child attachment and healthy human development. Routledge: Basic Books (1988).

[35] Janoff-Bulman R Shattered assumptions: towards a new psychology of trauma. New York NY, US: Free Press (1992).

[36] Wyrzykowska E Glogowska K Mickiewics K Attachment relationships among alcohol dependent persons. Alkoholizm i Narkomania (2014) 27(2):145–61. 10.1016/S0867-4361(14)70010-0

[37] Fairbairn CE Briley DA Kang D Fraley RC Hankin BL Ariss T A meta-analysis of longitudinal associations between substance use and interpersonal attachment security. Psychol Bull (2018) 144(5):532–55. 10.1037/bul0000141 PMC5912983 29494194

[38] Gidhagen Y Holmqvist R Philips B Attachment style among outpatients with substance use disorders in psychological treatment. Psychol Psychother (2018) 91(4):490–508. 10.1111/papt.12172 29399945

[39] Babapour Kheiroddin J Pursarifari H Soudmand M The role of therapeutic alliance and attachment styles in treatment drop-out among substance abusers. Res Addict Q J Drug Abuse (2018) 12(46):130–40.

[40] Min MO Tracy EM Park H Impact of trauma symptomatology on personal networks among substance using women. Drug Alcohol Depend (2014) 142. 10.1016/j.drugalcdep.2014.06.032 PMC4127087 25042762

[41] Strathearn L Maternal addiction: Does attachment play a role?. In: DMM NEWS., vol. 12 (2012). Retrieved from https://www.iasa-dmm.org/newsletter/view/march_2012_-_maternal_addiction_does_attachment_play_a_role/.

[42] Najavits LM Psychotherapies for trauma and substance abuse in women. Trauma, Violence & Abuse (2009) 10(3):290–8. 10.1177/1524838009334455 19477868

[43] Schindler A Bröning S A review on attachment and adolescent substance abuse: Empirical evidence and implications for prevention and treatment. Subst Abus (2015) 36:304–13. 10.1080/08897077.2014.983586 25424652

[44] Valizadeh M Motazedian S Kuchi MR Alipoor R Investigating the relationship between attachment styles and addiction severity. Bali Med J (2017) 6(2):68–73. 10.15562/bmj.v6i2.546

[45] De Palo F Capra N Simonelli A Salcuni S Di Riso D Parenting quality in drug-addicted mothers in a therapeutic mother–child community: The contribution of attachment and personality assessment. Front Psychol (2014) 5:1009. 10.3389/fpsyg.2014.01009 25309481 PMC4160036

[46] Slesnick N Feng X Brakenhoff B Brigham GS Parenting under the influence: The effects of opioids, alcohol and cocaine on mother–child interaction. Addict Behav (2014) 39:897–900. 10.1016/j.addbeh.2014.02.003 24589871 PMC4012539

[47] Porreca A Biringen Z Parolin M Saunders H Ballarotto G Simonelli A Emotional availability, neuropsychological functioning, and psychopathology: The context of parental substance use disorder. Biomed Res Int (2018) 5359037. 10.1155/2018/53590377 29888268 PMC5985126

[48] Tsantefski M Humphreys C Jackson AC Infant risk and safety in the context of maternal substance use. Child Youth Serv Rev (2014) 47:10–7. 10.1016/j.childyouth.2013.10.021

[49] Oji-Mmuo CN Corr TE Doheny KK Addictive disorders in women: the impact of maternal substance use on the fetus and newborn. NeoReviews (2017) 18(10):e576–86. 10.1542/neo.18-10-e576

[50] Bayley NA Diaz-Barbosa M Effect of maternal substance abuse on the fetus, neonate, and child. Pediat Rev (2018) 39(11):550–9. 10.1542/pir.2017-0201 30385584

[51] Konijnenberg C Sarfi M Melinder A Mother–child interaction and cognitive development in children prenatally exposed to methadone or buprenorphine. Early Hum Dev (2016) 101:91–7. 10.1016/j.earlhumdev.2016.08.013 27614330

[52] Kissin WB Svikis DS Morgan GD Haug NA Characterizing drugdependent women in treatment and their children. J Subst Abuse Treat (2001) 21:27–34. 10.1016/S0740-5472(01)00176-3 11516924

[53] Mayes L Truman S Substance abuse and parenting. In: Bornstein M, editor. Handbook of Parenting: Vol.4. Social Conditions and Applied Parenting., Lawrence Erlbaum (2002). p. 329–59.

[54] Neger EN Prinz RJ Interventions to Address Parenting and Parental Substance Abuse: Conceptual and Methodological Considerations. Clinical Psychology Review (2015) 39:71–82. 10.1016/j.cpr.2015.04.004 25939033 PMC4464898

[55] Håkansson U Söderström K Watten R Skårderud F Øie MG Parental reflective functioning and executive functioning in mothers with substance use disorder. Attach Hum Dev (2017) 20(2):181–207. 10.1080/14616734.2017.1398764 29105598

[56] Handeland TB Kristiansen VR Lau B Håkansson U Øie MG High degree of uncertain reflective functioning in mothers with substance use disorder. Addict Behav Rep (2019) 10. 10.1016/j.abrep.2019.100193 PMC6536670 31193539

[57] Barrocas J Vieira-Santos S Paixão R Parenting and drug addiction: a psychodynamic proposal based on a multifactorial perspective. Psychoanal Psychol (2016) 33(1):161–78. 10.1037/a0037344

[58] Davie-Gray A Moor S Spencer C Woodward LJ Psychosocial characteristics and poly-drug use of pregnant women enrolled in methadone maintenance treatment. Neurotoxicol Teratol (2018) 38:46–52. 10.1016/j.ntt.2013.04.006 23639593

[59] Li Z Lei K Coles CD Lynch ME Hu X Longitudinal changes of amygdala functional connectivity in adolescents prenatally exposed to cocaine. Drug Alcohol Depend (2019). 10.1016/j.drugalcdep.2019.03.007 PMC6607904 31085378

[60] Kim S Iyengar U Mayes LC Potenza MN Rutherford HJV Strathearn L Mothers with substance addictions show reduced reward responses when viewing their own infants’ face. Hum Brain Mapp (2017) 38:5421–39. 10.1002/hbm.23731 PMC5763911 28746733

[61] Bröning S Kumpfer K Kruse K Sack PM Schaunig-Busch I Ruths S Selective prevention programs for children from substance-affected families: a comprehensive systematic review. Substance Abuse, Treatment, Prevention, and Policy (2012) 7(1):23. 10.1186/1747-597X-7-23 PMC3490747 22691221

[62] Yule AM Wilens TE Martelon M Rosenthal L Biederman J Does exposure to parental substance use disorders increase offspring risk for a substance use disorder? A longitudinal follow-up study into young adulthood. Drug Alcohol Depend (2018) 186:154–8. 10.1016/j.drugalcdep.2018.01.021 PMC5876721 29573650

[63] Clark DB Cornelius JR Wood DS Vanyukov MM Psychopathology risk transmission in children of parents with substance use disorders. Am J Psychiatry (2004) 161(4):685–91. 10.1176/appi.ajp.161.4.685 PMC3155941 15056515

[64] Johnson JL Boney TY Brown BS Evidence of depressive symptoms in children of substance abusers. International Journal of the Addictions (1990) 25(4):465–79. 10.3109/10826089009105125 2093089

[65] Runtz M Godbout N Briere J Eadie E, (2014). Development of a new measure of adult relational attachment and its links with interpersonal trauma. Conference Paper.

[66] Fonagy P Conference paper. Alumni Conference “Twenty years of developmental lines”. In: Psychoanalysis and attachment theory: Need for a new integration? Anna Freud Centre. (2014).

[67] Fagin A Attending to Infant Mental Health. In: Smith L, editor. Clinical Practice at the Edge of Care. (2016). 10.1007/978-3-319-43570-1

[68] Musetti A Terrone G Corsano P Magnani B Salvatore S Exploring the link among state of mind concerning childhood attachment, attachment in close relationships, parental bonding and psychopathological symptoms in substance users. Front Psychol (2016) 7(1193). 10.3389/fpsyg.2016.01193 PMC4977822 27555832

[69] Grubac K Dimitrijevic A Hanak N Attachment and heroin addiction. Unpublished paper. Belgrade University: Faculty of Philosophy, Department of Psychology (2011).

[70] Eiden RD Maternal substance use and mother–infant feeding interactions. Infant Mental Health J (2001) 22(4):497–511. 10.1002/imhj.1013

[71] Söderstrom K Skårderud F Minding the baby. Mentalization-based treatment in families with parental substance use disorder: Theoretical framework. Nord Psychol (2009) 61(3):47–65. 10.1027/1901-2276.61.3.47

[72] Parolin M Simonelli A Attachment theory and maternal drug addiction: the contribution of parenting interventions. Front Psychiatry (2016) 7(152). 10.3389/fpsyt.2016.00152 PMC5004230 27625612

[73] Hatzis D Dawe S Harnett P Barlow J Quality of caregiving in mothers with illicit substance use: a systematic review and meta-analysis. Subst Abuse (2017) 11:1–15. 10.1177/1178221817694038 PMC5398331 28469425

[74] Felitti VJ Anda RF Nordenberg D Williamson DF Spitz AM Edwards V Relationship of childhood abuse and household dysfunction to many of the leading causes of death in adults: The Adverse Childhood Experiences (ACE) Study. American Journal of Preventive Medicine (1998) 14(4):245–58. 10.1016/S0749-3797(98)00017-8 9635069

[75] Rothman EF Edwards EM Heeren T Hingson RW Adverse childhood experiences predict earlier age of drinking onset: results from a representative US sample of current or former drinkers. Pediatrics (2008) 122(2):298–304. 10.1542/peds.2007-3412 18676515

[76] Arria AM Mericle AA Meyers K Winters KC Parental substance use impairment, parenting and substance use disorder risk. J Subst Abuse Treat (2012) 43(1):114–22. 10.1016/j.jsat.2011.10.001 PMC3289725 22112506

[77] Rusby JC Light JM Crowley R Wesling E Influence of parent-youth relationship, parental monitoring, and parent substance use on adolescent substance use onset. J Fam Psychol (2018) 32(3):310–20. 10.1037/fam0000350 PMC5920742 29300096

[78] Merrick MT Ports KA Ford DC Afifi TO Gershoff ET Grogan-Kaylor A Unpacking the impact of adverse childhood experiences on adult mental health. Child Abuse Negl (2017) 69:10–9. 10.1016/j.chiabu.2017.03.016 PMC6007802 28419887

[79] Minnes S Singer LT Humphrey-Wall R Satayathum S Psychosocial and behavioral factors related to the post-partum placements of infants born to cocaine-using women. Child Abuse Negl (2008) 32(3):353–66. 10.1016/j.chiabu.2007.12.002 PMC2867108 18374413

[80] Wong RS Tung KTS Cheng AW Shiu YK Wong WHS Tso WWY Disentangling the effects of exposure to maternal substance misuse and physical abuse and neglect on child behavioral problems. J Interpers Violence (2019). 10.1177/0886260519849661 31130045

[81] Hawley TL Halle TG Drasin RE Thomas NG Children of Addicted Mothers: Effects of the ‘Crack Epidemic’ on the Caregiving Environment and the Development of Preschoolers. American Journal of Orthopsychiatry (1995) 65(3):364–79. 10.1037/h0079693 7485422

[82] Finger B Jobin A Bernstein VJ Hans S Parenting contributors to early emerging problem behaviour in children of mothers in methadone maintenance treatment. Infant and Child Development (2017) 27(1). 10.1002/icd.2042

[83] Julian MM Muzik M Rosenblum HL Parenting in the context of trauma: Dyadic interventions for trauma-exposed parents and their young children. (2018) 10.1007/978-3-319-65724-0_9

[84] Sprang G Staton-Tindall M Gustman B Freer B Clark JJ Dye H The impact of trauma exposure on parenting stress in rural America. J Child Adolesc Trauma (2013) 6:287–300. 10.1080/19361521.2013.836585

[85] Christie H Hamilton-Gichritsis C Alves-Costa F Tomlinson M Halligan SL The impact of parental posttraumatic stress disorder on parenting: A systematic review. Eur J Psychotraumatol (2019) 10. 10.1080/20008198.2018.1550345 PMC6338266 30693071

[86] Fuchshuber J Hiebler-Ragger M Ragger K Kresse A Kapghammer HP Unterrainer HF Trauma, drive and disorder: the development of a psychodynamic model of substance abuse — preliminary findings. Grüner Kreis: Medical University of Graz (2017).

[87] Freisthler B Gruenewald PJ Wolf JP Examining the relationship between marijuana use, medical marijuana dispensaries, and abusive and neglectful parenting. Child Abuse Negl (2015) 48. 10.1016/j.chiabu.2015.07.008 PMC4593739 26198452

[88] Freisthler B Kepple NJ Wolf JP Curry SR Gregoire T Substance use behaviors by parents and the decision to substantiate child physical abuse and neglect by caseworkers. Child Youth Serv Rev (2017) 79:576–83. 10.1016/j.childyouth.2017.07.014

[89] Kepple NJ Does parental substance use always engender risk for children? Comparing incidence rate ratios of abusive and neglectful behaviors across substance use behavior patterns. Child Abuse Negl (2018) 76:44–55. 10.1016/j.chiabu.2017.09.015 29032186

[90] Cross D Ruchard A Jovanovic T Vance LA Kim YJ Fox N Trauma exposure, PTSD, and parenting in a community sample of lowincome, predominantly African American mothers and children. Psychol Trauma (2017). 10.1037/tra000264 PMC5677577 28481561

[91] Egeland B Jacobvitz D Papatola K Intergenerational conrinuity of abuse. In: Gelles RJ Lan- caster JB, editors. Child Abuse and Neglect: Biosocial Dimensions. Aldine de Gruyter (1987). p. 255–76.

[92] Farisco M Evers K Changeux JP Drug Addiction: From Neuroscience to Ethics. Frontiers in Psychiatry (2018) 9(595). 10.3389/fpsyt.2018.00595 PMC6262362 30524319

[93] Melo MC Corradi-Webster CM Meanings about mothering by women in treatment for drug use. Estud. Psicol. (Campinas) (2016) 33(4):699–709. 10.1590/1982-02752016000400013

[94] Barlow J Sembi S Parsons H Kim S Petrou S Harnett P A randomized controlled trial and economic evaluation of the Parents under Pressure Program for parents in substance abuse treatment. Drug and Alcohol Dependence (2019) 194:184–94. 10.1016/j.drugalcdep.2018.08.044 30447510

[95] Srivastava P Hopwood N Reflection/commentary on a past article: “A practical iterative framework for qualitative data analysis”. Int J Qual Methods (2018) 17:1–3. 10.1177/1609406918788204

[96] Aspers P Corte U What is qualitative in qualitative research. Qual Sociol (2019) 42(2):139–60. 10.1007/s11133-019-9413-7 PMC6494783 31105362

[97] Adriansen HK Timeline interviews: A tool for conducting life history research. Qual Stud (2012) 3(1):40–55. 10.7146/qs.v3i1.6272

[98] Castleberry A Nolen A Thematic analysis of qualitative research data: Is it as easy as it sounds? Curr Pharm Teach Learn (2018) 10(6):807–15. 10.1016/j.cptl.2018.03.019 30025784

[99] Braun V Clarke V Using thematic analysis in psychology. Qual Res Psychol (2006) 3(2):77–101. 10.1191/1478088706qp063oa

[100] Braun V Clarke V Thematic analysis. In: Cooper H, editors. APA Handbook of Research Methods in Psychology., vol. 2 American Psychological Association (2012). p. 57–71. 10.1037/13620-004

[101] Padykula LFN Conklin P The self-regulation model of attachment trauma and addiction. Clin Soc Work J (2010) 38:351–60. 10.1007/s10615-009-0204-6

[102] Cihan A Winstead DA Laulis J Feit MD Attachment theory and substance abuse: Ethiological links. J Hum Behav Soc Environ (2014) 24(5). 10.1080/10911359.2014.908592

[103] Fletcher K Nutton J Brend DM Attachment, a matter of substance: The potential of attachment theory in the treatment of addictions. Clinical Social Work Journal (2015) 43(1):109–17. 10.1007/s10615-014-0502-5

[104] MacLean P The triune brain in evolution: Role in paleocerebral functions. New York: Plenum (1990). 10.1126/science.250.4978.303-a 17797318

[105] Reading B The application of Bowlby’s attachment theory to the psychotherapy of addiction. In: Weegman M Cohen R, editors. Psychodynamics of addiction. John Wiley and Sons. Ltd. (2002). p. 13–30. 10.1002/9780470713655.ch2

[106] Fletcher K Nutton J Brend D Attachment, a matter of substance: The potential of attachment theory in the treatment of addictions. Clin Soc Work J (2014) 10.1007/s10615-014-0502-5

[107] Klein S Addiction and attachment: A complex relationship. Clin Neurosci (2013) .

[108] Shults C Addiction and Attachment. In: DMM NEWS., vol. 12 (2012).

[109] Gill R Addictions from an attachment perspective: Do broken bonds and early trauma lead to addictive behavior. New York, NY: Routledge (2017).

[110] Levy MS A helpful way to conceptualize and understand reenactments. J Psychother Pract Res (1998) 7(3):227–35. PMC3330499 9631344

[111] Khantzian EJ The theory of self-medication and addiction. Psychiatric Times (2017) 34(2).

[112] Sweet AD Aspects of internal self and object representations in disorganized attachment: Clinical considerations in the assessment and treatment of chronic and relapsing substance misusers. British Journal of Psychotherapy (2013) 29(2):154–67. 10.1111/bjp.12013

[113] Mikulincer M Shaver PR Solomon Z An attachment perspective on traumatic and posttraumatic reactions. (2015). 10.1007/978-1-4899-7522-5_4

[114] Bakhshani NM Hossienbor M A Comparative Study of Self-Regulation in Substance Dependent and Non-Dependent Individuals. Global Journal of Health Science (2013) 5(6):40–5. 10.5539/gjhs.v5n6p40 PMC4776852 24171872

[115] Baumeister RF Vohs KD Self-regulation, ego depletion, and motivation. Social and Personality Psychology Compass (2007) 1(1):115–28. 10.1111/j.1751-9004.2007.00001.x

[116] Garami J Valikhani A Parkes D Haber P Mahlberg J Misiak B Examining perceived stress, childhood trauma and interpersonal trauma in individuals with drug addiction. Psychol Rep (2018) 0(0):1–18. 10.1177/0033294118764918 29569991

[117] Weegmann M Khantzian EJ Dangerous desires and inanimate attachments. In: Modern psychodynamic approaches to substance misuse. (2017).

[118] Iyengar U Rajhans P Fonagy P Strathearn L Kim S Unresolved trauma and reorganization in mothers: Attachment and neuroscience perspectives. Front Psychol (2019) 10(110). 10.3389/fpsyg.2019.00110 PMC6363675 30761051

[119] McLaughlin A Campbell A McColgan M Adolescent substance use in the context of the family: a qualitative study of young people’s views on parent-child attachments, parenting style and parental substance use. Substance Use and Misuse (2016) (51):1846–55. 10.1080/10826084.2016.1197941 27606719

[120] Iyengar U Kim S Martinez S Fonagy P Strathearn L Unresolved trauma in mothers: intergenerational effects and the role of reorganization. Front Psychol (2014) 6(131). 10.3389/fpsyt.2015.00131 PMC4150444 25225490

[121] Miller S Klockner K Attachment styles and attachment based change in offenders in a prison Therapeutic Community. J Forensic Psychol Res Pract (2019). 10.1080/24732850.2019.1603956

[122] Bortolini M Piccinini CA Intergenerational transmission of secure attachment: Evidences from two cases. Psicologia em Estudo (2015) 20(2):247–59. 10.4025/psicolestud.v20i2.25246

[123] Bernard K Frost A Jelinek C Dozier M Secure attachment predicts lower body mass index in young children with histories of child protective services involvement. Pediatr Obes (2019). 10.1111/ijpo.12510 30659782

[124] Cornellà-Font MG Viñas-Poch F Juárez-López JR Martín-Perpiñá M de las M Malo-Cerrato S Temperament and attachment as predictive factors for the risk of addiction to substances in adolescents. Rev Psicopatol Psicol Clín (2018) 23(3):179–87. 10.5944/rppc.vol.23

[125] Streeck-Fischer A van der Kolk BA Down will come baby, cradle and all: diagnostic and therapeutic implications of chronic trauma on child development. Chronic Trauma Child Devt (2000) 903–18. 10.1080/000486700265 11127621

[126] Vanderzee KL John SG Edge N Pemberton JR Kramer TL A preliminary evaluation of the managing youth trauma effectively program for substance-abusing women and their children. Infant Mental Health Journal (2017) 38(3):422–33. 10.1002/imhj.21639 28464299

[127] Tedgard E Rastam M Wirtberg I Struggling with one’s own parenting after an upbringing with substance abusing parents. Int J Qual Stud Health Well-being (2018) 13. 10.1080/17482631.2018.1435100 PMC5827643 29482480

[128] Chamberlain C Gee G Harfield S Campbell S Brennan S Clark Y Parenting after a history of childhood maltreatment: a scoping review and map of evidence in the perinatal period. PLoS One (2019) 14(3). 10.1371/journal.pone.0213460 PMC6415835 30865679

[129] Finkelstein J Yates JK Traumatic symptomatology in children who witness marital violence. Intl J Emerg Mental Health (2001) 3(2):107–14. 11508563

[130] Pynoos M Nader K Children who witness the sexual assaults of their mothers. J Am Acad Child Adolesc Psychiatry (1988) 27(5):567–72. 10.1097/00004583-198809000-00009 3182620

[131] Andersen SL Stress, sensitive periods, and substance abuse. Neurobiol Stress (2019) 10. 10.1016/j.ynstr.2018.100140 PMC6288983 30569003

[132] German M Umylny P Mason Z Schrag R Silver E Krug L Poster presentation at the Society for Developmental and Behavioral Pediatrics Annual Meeting. In: Early identification of children at greatest risk: How do we assess parental trauma? TN. (2014).

[133] Salberg J The texture of traumatic attachment: Presence and ghostly absence in transgenerational transmission. Psychoanal Q (2015) LXXXIV(1):21–46. 10.1002/j.2167-4086.2015.00002.x 25619365

[134] Brothers D Traumatic attachments: intergenerational trauma, dissociation, and the analytic relationship. Intl J Psychoanal Self Psychol (2013) 9(1). 10.1080/15551024.2014.857746

[135] Balbernie R All about … Intergenerational trauma. Nursery World (2017), 24–7. 10.12968/nuwa.2017.17.24

[136] Yang C Xia M Zhou Y The relationship between self-control and selfefficacy among patients with substance use disorders: Resilience and selfesteem as mediators. Front Psychiatry (2019). 10.3389/fpsyt.2019.00388 PMC6582546 31249535

[137] Köpetz CE Lejuez CW Wiers RW Kruglanski AW Motivation and self-regulation in addiction: a call for convergence. Perspect Psychol Sci (2013) 8(1):3–24. 10.1177/1745691612457575 26069472 PMC4461059

[138] Hine RH Maybery DJ Goodyear MJ Identity in personal recovery for mothers with a mental illness. Front Psychiatry (2019) 10(89). 10.3389/fpsyt.2019.00089 PMC6418025 30906268

[139] Markus W de Kruijk C de Weert-van Oene GH Becker ES De Jong CA One size fits few: Een pleidooi voor maatwerk bij geïntegreerd behandelen van PTSS en verslaving. Directieve Therapie en Hypnose (2014) 34(3):180–201.

[140] Isobel S Goodyear M Furness T Foster K Preventing intergenerational trauma transmission: A critical interpretive synthesis. J Clin Nurs (2018) 1–14. 10.1111/jocn.14735 30556334

[141] Van den Brink W Substance use disorders, trauma, and PTSD. European Journal of Psychotraumatology (2015) 6(1):27632. 10.3402/ejpt.v6.27632

[142] Missouridou E Cultivating a trauma awareness culture in the addictions. Curr Drug Abuse Rev (2017) 9(2). 10.2174/1874473710666170111102835 28078986

[143] Lotzin A Grundmann J Hiller P Pawils S Schäfer I Profiles of childhood trauma in women with substance use disorders and comorbid posttraumatic stress disorders. Front Psychiatry (2019) 10.3389/fpsyt.2019.00674 PMC6813657 31681026

[144] Cruise KE Becerra R Alexithymia and problematic alcohol use: a critical update. Addict Behav (2018) 77:232–46. 10.1016/j.addbeh.2017.09.025 29107201

[145] Hamidia S Rostami R Farhoodia F Abdolmanafia A A study and comparison of alexithymia among patients with substance use disorder and normal people. Procedia Soc Behav Sci (2010) 5:1367–70. 10.1016/j.sbspro.2010.07.289

[146] Thorberg FA Alexithymia, craving and attachment in a heavy drinking population. Addict Behav (2011) 36(4):427–30. 10.1016/j.addbeh.2010.12.016 21215527

[147] Besharat MA Khajavi Z The relationship between attachment styles and alexithymia: mediating role of defense mechanisms. Asian J Psychiatry (2013) 6:571–6. 10.1016/j.ajp.2013.09.003 24309875

[148] Boisjoli C Hébert M Gauthier-Duchesne A Caron P A mediational model linking perceptions of security, alexithymia and behavior problems of sexually abused children. Child Abuse Negl (2019) 92:66–76. 10.1016/j.chiabu.2019.03.017 30933832

[149] Kefeli MC Turow RG Yıldırım A Boysan M Childhood maltreatment is associated with attachment insecurities, dissociation and alexithymia in bipolar disorder. Psychiatry Res (2018) 260:391–9. 10.1016/j.psychres.2017.12.026 29253803

[150] Davey S Halbertstadt J Bell E Collings S A scoping review of suicidality and alexithymia: The need to consider interoception. J Affect Disord (2018) 238:424–41. 10.1016/j.jad.2018.06.027 29913380

[151] De Berardis D Fornaro M Orsolini L Valchera A Carano A Vellante F Alexithymia and suicide risk in psychiatric disorders: A mini-review. Front Psychiatry (2017) 8(148). 10.3389/fpsyt.2017.00148 PMC5557776 28855878

[152] De Berardis D Fornaro M Valchera A Rapini G Di Natale S De Lauretis I Alexithymia, resilience, somatic sensations and their relationships with suicide ideation in drug naïve patients with first-episode major depression: An exploratory study in the “real world” everyday clinical practice. Early Interv Psychiatry (2019). 10.1111/eip12863 31402575

