# Supplementary material for: Mothering, Substance Use Disorders and Intergenerational Trauma Transmission: An Attachment-Based Perspective
Source: Front Psychiatry. 2019 Oct 18;10:728. doi: 10.3389/fpsyt.2019.00728 (PMC6813727; doi:10.3389/fpsyt.2019.00728)
Supplement: Supplementary file 1 [file Table_1.docx]

TABLE S1: Participants’ characteristics (n = 23)

Table 1

*Participants’ characteristics (n = 23)*

| **Characteristic** | **Value** | **n = 23** |
| --- | --- | --- |
| **Ethnicity** | Western European  South Slavic | 22  1 |
| **Relationship status** | Single or divorced  Cohabitating  Incarcerated partner | 14  8  1 |
| **Number of children** | 1  2  3  5 | 11  6  5  1 |
| **Primary substance of choice at the time of upbringing** | Amphetamine  Cannabis  Cocaine  Heroin  GHB | 8  6  5  3  1 |
| **Current substance use status** | Clean  Active use | 13  10 |
| **Treatment modality** | **Inpatient treatment programs**  Medical detoxification  Short-term residential treatment^1^  Long-term residential treatment^2^  **Outpatient treatment programs**  Substitution treatment (e.g. methadone maintenance) + individual counseling  Pharmacological based treatment and/or individual counseling^3^ | **11**  2  3  6  **12**  2  10 |
| **Number of mothers who lost custody of their children** | Voluntarily assisted by child protection services  Court-ordered measure | 4  15 |
| ^1^ group therapy, lengths of stay between 4 and 6 weeks  ^2^ group therapy, lengths of stay between 6 and 12 months ^3^ twice-weekly or on a monthly basis, often with breaks in between |  |  |
